# Supporting the “hallway residents”: a qualitative descriptive study of staff perspectives on implementing the Namaste Care intervention in long-term care

**DOI:** 10.1186/s12877-023-04360-9

**Published:** 2023-10-16

**Authors:** Donny H.Y. Li, Marie-Lee Yous, Paulette V. Hunter, Esther Coker, Danielle Just, Vanina Dal Bello-Haas, Carrie McAiney, Abigail Wickson-Griffiths, Sharon Kaasalainen

**Affiliations:** 1 School of Nursing, Faculty of Health Sciences, McMaster University, Hamilton, ON Canada; 2 Michael G. DeGroote School of Medicine, Faculty of Health Sciences, McMaster University, Hamilton, ON Canada; 3 Department of Psychology, St. Thomas More College, University of Saskatchewan, Saskatoon, SK Canada; 4 St. Peter’s Hospital, Hamilton Health Sciences, Hamilton, ON Canada; 5 Lawrence S. Bloomberg Faculty of Nursing, University of Toronto, Toronto, ON Canada; 6 School of Rehabilitation Science, Faculty of Health Sciences, McMaster University, Hamilton, ON Canada; 7 School of Public Health Sciences, University of Waterloo and Schlegel-UW Research Institute for Aging, Waterloo, ON Canada; 8 Faculty of Nursing, University of Regina, Regina, SK Canada

**Keywords:** Advanced dementia, Social isolation, Long-term care, Namaste Care, Quality of life

## Abstract

**Background:**

Long-term care (LTC) settings are becoming home to an increasing number of people living with advanced or late-stage dementia. Residents living with advanced dementia represent some of society’s most vulnerable and socially excluded populations and are thus at an increased risk of social isolation. A multisensory intervention tailored to this population, *Namaste Care*, has been developed to improve quality of life for residents living with advanced dementia in LTC homes. To date, limited research has explored the perspectives of staff in implementing the Namaste Care program with an emphasis on social inclusion of residents in Canadian LTC homes. This study aimed to describe the perspectives of LTC staff on the implementation facilitators and barriers of Namaste Care as a program to support the social inclusion of residents living with advanced dementia.

**Methods:**

Using a qualitative descriptive design, semi-structured interviews (n = 12) and focus groups (n = 6) were conducted in two LTC homes in Southern Ontario, Canada, over a 6-month period. Convenience sampling was used to recruit LTC home staff from the two participating sites. Thematic analysis was used to analyze data.

**Results:**

LTC staff (n = 46) emphasized the program’s ability to recognize the unique needs of residents with advanced dementia, and also stated its potential to facilitate meaningful connections between families and residents, as well as foster care partnerships between staff and families. Findings indicated staff also perceived numerous facilitators and barriers to Namaste Care. In particular, providing staff with dedicated time for Namaste Care and implementing volunteer and family participation in the program were seen as facilitators, whereas the initial perception of the need for extra staff to deliver Namaste Care and identifying times in the day where Namaste Care was feasible for residents, families, and staff, were seen as barriers.

**Conclusions:**

LTC staff recognized the need for formalized programs like Namaste Care to address the biopsychosocial needs of residents with advanced dementia and offer positive care partnership opportunities between staff and family members. Although staffing constraints remain the largest barrier to effective implementation, staff valued the program and made suggestions to build LTC home capacity for Namaste Care.

## Background

The number of Canadians living with dementia is predicted to reach 1.7 million by 2050, a significant increase from 2020 when 597,000 Canadians were living with dementia [1]. Of the over half a million Canadians currently living with dementia, approximately 31% of those between the ages of 65–79 and 42% of those over the age of 80 live in long-term care (LTC) [2, 3]. These individuals are among those most at risk for social isolation, with those living with advanced (i.e., late-stage) dementia at an increased risk [4]. Social isolation may lead to depressive symptoms, more frequent and severe pain, and an overall decrease in quality of life [5] and has been identified as a global health crisis among older adults by numerous reports [6, 7]. Thus, there is a compelling need for work to promote social and meaningful engagement, particularly for people living with advanced dementia, in LTC [7–10].

Advanced dementia is the end of the disease trajectory for dementia. At this stage, people living with dementia are typically unable to communicate or plan other motor behaviours effectively and require care 24 hours a day [11]. An important goal of care at this stage is to enhance quality of life for the person and attend to the psychosocial needs of their family [11]. Given the decline in communication ability by this stage, families and staff may experience challenges in adapting to quality of life promoting activities or social interactions, and this can further contribute to social isolation [12, 13].

Social isolation is defined as a low quality and quantity of contact with others, resulting in few social connections or social roles, and a decreased or non-existent number of mutually rewarding relationships [4]. In particular, social isolation in LTC residents often means a sense of separation and loss of personal connection with family and friends outside the home [14–16]. Most people living with dementia in LTC homes in Canada are supported by family caregivers providing care (e.g., socialization, nutritional assistance, hygiene care) [17] with 64% of caregivers spending up to ten hours a week and 21% of caregivers spending twenty hours or more [17]. Yet, as persons with dementia progress towards more advanced stages, some family caregivers may benefit from additional support and education to meaningfully engage with relatives who have dementia. This can be especially important for these family members as caregiver burden can be a significant problem for caregivers of older adults with a chronic condition; family caregivers may progressively become vulnerable to social isolation, emotional and social challenges, and lower quality of life [18, 19]. A strong need exists in LTC for an intervention that supports the social connection, well-being, and meaningful engagement of residents at all stages, [20] especially among those on the dementia spectrum and their family carers.

In response to this need, a person-centered, multisensory approach to support persons living with dementia called *Namaste Care* was developed by Joyce Simard and Ladislav Volicer to facilitate social connections and meaningful engagement for residents who live in LTC [21]. Namaste Care was developed to benefit residents living with advanced dementia, who are no longer able to participate in traditional social programs or activities such as exercise groups, crafts, and games [21]. Namaste Care is based on the principle that persons living with advanced dementia can still benefit from meaningful activities that affirm their personhood and enhance their quality of life when those activities are properly adapted and supported by carers, including LTC staff and families [21, 22]. To date, several studies have investigated the benefits associated with Namaste Care for residents living with advanced dementia in LTC. In the United Kingdom, LTC homes found that the Namaste Care program decreased the responsive behaviours of residents living with advanced dementia with the support of strong leadership and adequate nursing care [21, 23]. A more recent study found evidence to support Namaste Care as an intervention to improve the quality of life of residents living with advanced dementia in LTC, which subsequently had a positive impact on the well-being of family carers [24]. Furthermore, Namaste Care has been shown to decrease the use of anti-anxiety medication for residents [25]. Findings from a Canadian mixed method study found that Namaste Care decreased resident pain level and improved quality of life [26]. There is also some evidence from a cluster-randomized controlled trial to suggest that Namaste Care is also more cost-effective when compared to usual care in residents with advanced dementia [27].

While numerous studies have explored questions concerning the benefits of the Namaste Care program in LTC, relatively few have inquired about staff perspectives on the implementation facilitators and barriers of the program in Canada, especially from LTC staff in various disciplines, such as those in management roles [26, 28]. There is also a need to explore staff moral achievement in LTC in Canada which can occur when staff recognize gaps in care for persons with dementia and facilitate activities and services to support their social inclusion. Through this ethical stance towards care provision, participation of all staff members is required and becomes a professional responsibility to enhance the experience of care [29]. The aim of this study was to describe the perspectives of LTC home staff on the implementation facilitators and barriers of Namaste Care as a program to support the social inclusion of residents living with advanced dementia. This study was a sub-study of a larger 4-year mixed methods study aimed at evaluating the Namaste Care program as a strategy to improve social inclusion of people with dementia and their family carers.

## Methods

### Design

A qualitative description approach was used to describe the perspectives of LTC home staff on the Namaste Care intervention for residents living with advanced dementia in LTC following its implementation [30].

### Namaste Care Intervention

Namaste Care sessions were facilitated by a staff carer (e.g., personal support workers, nurses, or activity aides) with the support of volunteers. The Namaste Care program was expected to run 5–7 days/week, 2–4 h/day in the morning and afternoon in dedicated rooms and within a calming, multi-sensory environment (e.g., music, scents, lighting, comfortable chairs with blankets). Family involvement was encouraged (e.g., applying hand cream, brushing hair, assisting with nourishments). In the Namaste room, soft lighting is provided, gentle music is played, and the scent of lavender is diffused. A small group of residents are positioned in comfortable reclining chairs. Snacks and beverages are offered by staff carers, family members, or volunteers for comfort and to provide residents with nourishment and hydration. Activities are tailored to the preferences and life stories of residents. A total of 53 residents and 42 family carers participated in the Namaste Care program. The mean number of weeks a resident was enrolled in Namaste Care was 24.86 (standard deviation (SD) = 2.80) out of 26 weeks. On average, each resident attended two Namaste Care sessions each week which did not meet the target of at least 10/14 sessions (80%) per week. More information about program delivery is available elsewhere [31].

### Sample and Setting

Participants from two not-for-profit LTC homes in urban areas of Southern Ontario, Canada, were recruited; sites one and two had 127 and 210 beds, respectively. At the time of focus groups and interviews, both homes had implemented Namaste Care for approximately three to six months. Through convenience sampling, study participants were recruited from the two study sites based on recommendations made by the senior leadership staff and advertisement at team meetings. These recommendations were based on who the leadership staff at the home believed had sufficient knowledge in the Namaste Care intervention at the home and who would be interested in participating in the study. Participants were also required to meet the following inclusion criteria: English-speaking, employed at one of the two study sites (LTC home), experience with residents living with advanced dementia, and involvement in the Namaste Care program. The sample size was deemed sufficient once diverse staff perspectives were included and overlapping data supported data saturation.

### Data Collection and Analysis

Data were collected over a 6-month period between November 2017 and April 2018 using semi-structured interviews and focus groups conducted by five research assistants who received training in qualitative interviewing. These research assistants had a background in nursing, psychology, and health sciences. They underwent initial in-person training on data collection with resources on how to conduct focus groups and interviews followed by additional training by the research coordinator if needed. Demographic data were collected prior to implementing Namaste Care using a questionnaire that asked participants about their age, sex, current role, years of experience in a field related to dementia, and years of experience in their current LTC home. Interviews were conducted with home leadership (management) participants. Home leadership participants took part in interviews rather than focus groups to ensure that staff would feel comfortable in sharing their opinions without the presence of management staff. Interviews were conducted over the phone due to the preferences of participants and to accommodate their schedules. One series of focus groups were conducted separately for caregiving staff of the same discipline such as nurses and personal support workers (PSWs). Focus groups were completed in person at the study sites. Other staff (i.e., nutrition manager, recreation programmer, maintenance servicer, housekeeping staff) were grouped together due to lower numbers of staff in these roles. Focus groups were conducted based on disciplines when possible as participants from the same discipline could contribute to richer dialogue due to shared understandings. Interview and focus group guides were developed through a search of studies exploring concepts such as Namaste Care and its implementation, conducted by the principal investigator and research assistants. In general, these interviews and focus groups aimed to capture staff’s current home practices and challenges regarding care for patients with dementia, as well as their evaluation of and experience with the Namaste Care program. Interviews and focus group sessions varied in length, from roughly twenty minutes to one hour.

Examples of questions asked at the focus groups and interviews were: (a) What was your experience implementing Namaste Care; (b) What factors helped the program run at [name of LTC home]; (c) What were the challenges experienced in running the program; and (d) How did Namaste Care create opportunities for you to socially engage with residents with dementia more than you normally would in your role? For focus groups in particular, the discussions were guided by semi-structured questions asking participants to describe care delivery for persons with advanced dementia such as how care was being provided for persons with advanced dementia prior to Namaste Care, how families are engaged in the care of persons with advanced dementia, how Namaste Care is being delivered at the home, how families are engaged in the program, and facilitators and barriers to implementing Namaste Care. There were one to two research assistants facilitating each focus group and they ensured that all members contributed to discussions. Refreshments were provided for study participants at the in-person focus groups. All participants were provided a small honorarium in the amount of a $25 CAD gift card to acknowledge their time and contributions to the study. All interviews and focus groups were audio recorded using a digital recorder, and verbatim transcripts were generated by a professional transcriptionist; thematic analysis was then employed [32].

Data analysis was conducted by the principal investigator and research assistants, who all identified as female. The principal investigator (SK) is a senior researcher with qualitative research experience and the research assistants completed post-secondary courses in qualitative research. Two research assistants reviewed the transcripts prior to performing initial coding. Using deductive analysis, interview data was assessed based on a predefined set of categories, which included implementation facilitators and barriers. The coding structure that focused on the categories of implementation facilitators and barriers was reviewed with the principal investigator and research assistants before sorting data in the two categories. Afterwards, the team coded data under each category. For example, under implementation facilitators, we developed codes that reflected different groups of individuals involved in implementing Namaste Care, such as staff, families, and volunteers, examining how their involvement helped to provide support for program delivery. Finally, we merged codes under each category into themes. We focused on commonalities to determine themes such as encouraging family involvement as an implementation facilitator. Monthly team meetings were held to discuss themes to ensure that they fit within the coding structure. Demographic data associated with study participants were summarized using mean and standard deviation for continuous variables, and counts/percentages for categorical variables.

### Rigour and Trustworthiness

To enhance rigour and trustworthiness in qualitative research, the research team (DL, MY, PVH, EC, DJ, VDBH, CM, AWG, SK) attended to Lincoln and Guba’s trustworthiness criteria: credibility, transferability, dependability, and confirmability [33]. Investigator triangulation was used to ensure credibility of findings by seeking feedback from all members of the research team who have expertise in LTC, palliative care, and dementia care contexts. In particular, the analysts invited all members of the team to reflect on preliminary findings, and this input was helpful in framing the analysis. Joint meetings were held with members of the research team every two months. Detailed descriptions of the setting and study sample were provided to increase the transferability of findings [33]. With regards to dependability and confirmability, the research team ensured that study processes were logically sound by conducting a comprehensive review of the existing literature on the social inclusion of people with advanced dementia, and on the availability of psychosocial interventions for persons with advanced dementia in LTC, including intervention implementation facilitators and barriers. An audit was also conducted to ensure that the study processes adhered to the protocol, and research assistants made field notes after data collection.

## Findings

### Characteristics of the Sample

The 46 LTC staff who participated in the semi-structured interviews and focus groups held various roles in LTC. Demographics were provided by all but five staff members. Based on this information, there were 32 female and 7 male participants. One participant identified as another gender not specified by the questionnaire, and another did not respond. The age of study participants varied. Prior to implementing Namaste Care, study participants had a mean of 10.98 years (SD = 8.99) of experience working in a field related to dementia, with 54% reporting that they were ‘very involved’ in dementia care, 32% reporting ‘somewhat involved,’ and 10% reporting ‘not involved at all’. See Table 1 for additional demographic characteristics.

**Table 1:** Demographic characteristics of staff participants (n = 41)

Characteristics	*n* (%) (Site 1)	*n* (%) (Site 2)
Age in years		
Under 25	2 (8.7)	0 (0.0)
25–34	5 (21.7)	3 (16.7)
35–44	7 (30.4)	3 (16.7)
45–54	5 (21.7)	7 (38.9)
55–64	3 (13.0)	5 (27.8)
No response	1 (4.3)	0 (0.0)
**Gender**		
Male	4 (17.4)	3 (16.7)
Female	17 (73.9)	15 (83.3)
Another Gender Not Specified	1 (4.3)	0 (0.0)
No response	1 (4.3)	0 (0.0)
**Current role(s)**		
Director of Care	2 (8.7)	1 (5.6)
Registered Nurse	2 (8.7)	2 (11.1)
Registered Practical Nurse	6 (26.1)	3 (16.7)
Personal Support Worker	6 (26.1)	5 (27.8)
Recreation Staff	4 (17.4)	1 (5.6)
Housekeeper or Cleaner	0 (0.0)	2 (11.1)
Cook or Kitchen Staff	0 (0.0)	1 (5.6)
Other (e.g., Student Registered Practical Nurse, Maintenance, Life Transitions Coach, Coordinator of Volunteers, Resident Care Supervisor, Administrator)	3 (13.0)	3 (16.7)
**Employment status**		
Part-Time	6 (26.1)	5 (27.8)
Full-Time	17 (73.9)	13 (72.2)
**Years of experience in field related to dementia**		
0–2	6 (26.1)	1 (5.6)
3–5	7 (30.4)	2 (11.1)
6–10	4 (17.4)	6 (33.3)
11–15	1 (4.3)	3 (16.7)
Over 15	4 (17.4)	6 (33.3)
No Response	1 (4.3)	0 (0.0)
**Years of experience in current LTC home**		
0–2	9 (39.1)	4 (22.2)
3–5	4 (17.4)	1 (5.6)
6–10	4 (17.4)	7 (38.9)
11–15	2 (8.7)	6 (33.3)
Over 15	4 (17.4)	0 (0.0)
**Received training specific to dementia care**		
No	0 (0.0)	2 (11.1)
Yes	23 (100.0)	16 (88.9)
**Level of involvement in the planning or delivery of care for residents with dementia prior to Namaste Care**		
Not Involved at All	1 (4.3)	3 (16.7)
Somewhat Involved	9 (39.1)	4 (22.2)
Very Involved	11 (47.8)	11 (61.1)
No response	2 (8.7)	0 (0.0)

### Overview of Findings

Themes were grouped under Namaste Care implementation facilitators and barriers in LTC. Study IDs were generated using an algorithm across both sites and used as identifiers for quotes. UM (unit manager), NUR (nurse), PSW (personal support worker), and LTCS (LTC staff). Unit managers consisted of Directors of Care, nurses included Registered Nurses and Registered Practical Nurses, and LTC staff included Recreational Staff, Housekeepers or Cleaners, Cook or Kitchen Staff, and other staff (e.g., Student Registered Practical Nurse, Maintenance, Life Transitions Coach, Coordinator of Volunteers, Resident Care Supervisor, Administrator). See Table 2 for an overview of themes.

**Table 2:** Categories and themes of LTC home staff perspectives on the Namaste Care Program

Categories	Themes
1. Implementation Facilitators	a) Providing dedicated staff time
	b) Recruiting volunteer assistance
	c) Encouraging family involvement
2. Implementation Barriers	a) Low staffing ratios
	b) Timing of Namaste Care sessions for families

### Implementation Facilitators

With regards to implementation facilitators, participants perceived that many facilitators were put in place to support the implementation of Namaste Care. Namaste Care was perceived as best delivered by: (a) providing dedicated staff time; (b) recruiting volunteer assistance; and (c) encouraging family involvement.

*Providing dedicated staff time.* PSWs and other LTC staff identified Namaste Care as a means to support their own social connections and meaningful engagement with residents living with advanced dementia. Staff members who delivered the program found it helpful to be provided with a block of time to deliver Namaste Care for residents and focus on providing meaningful engagement. They stated that Namaste Care facilitates their ability to do the *“extra little things”* (1PSW 4 Focus Group) and provide the opportunity for more personal time with residents. In providing care in LTC, PSWs referenced:

*“It’s an assembly line. This resident, this resident…there’s no downtime in between to just take that extra five minutes…[in Namaste Care], you get more personal one on one time with each [resident] if you’re…providing therapeutic touch for them. You get the one-on-one personal time with them. Because we…on a regular shift, we don’t have that time”* (1PSW 4 Focus Group).

Participants in management roles perceived that traditional programs (e.g., bingo, card games) offered in LTC are designed for residents with early to moderate dementia and staff previously had no dedicated time to engage with residents with advanced dementia. One UM participant stated that *“We would do our typical board games and things like that. But of course, the folks that are further along and have…severe Dementia or Alzheimer’s, they can’t participate in those things”* (1 UM 9). Thus, residents with advanced dementia have diverse needs and abilities that were found to be supported through Namaste Care.

Management participants strongly indicated that by allocating time for staff to support the *“hard to reach residents”* (1 UM 6), residents living with advanced dementia are not left *“sitting in the hallways”* (1 UM 5) but are instead offered meaningful activities adapted to their level of physical and cognitive abilities. Namaste Care was viewed as being able to fill a gap in the current programming of LTC homes. Implementing such a program was considered important to *“give them [residents with advanced dementia] a purpose”* (1 UM 9) and promote social inclusion.

Further, relationships between residents and staff were strengthened through Namaste Care, as PWSs stated that *“the more you know them [residents living with advanced dementia], the easier it is to work with them”* (1PSW 4 Focus Group). These quotes demonstrate that oftentimes, staff may not have the opportunity to fully understand and learn about their residents, though they have a strong desire to do so. Therefore, Namaste Care helps to slow down the pace of traditional shift work to provide a dedicated period for staff to meaningfully engage with residents with advanced dementia. This allows them to have an opportunity in providing such a type of care they may otherwise not have had a chance to engage in.

*Recruiting volunteer assistance.* Management staff identified volunteer help as one of the primary recommendations for implementing the Namaste Care program. Staff also noted that volunteers benefit by feeling valued by the difference they make:

*“[Volunteers] love the program and it’s what they come in to volunteer and do. Their passion is…they enjoy it, they love it, they love giving that individual care and they feel like they’re making a difference in each resident’s life”* (1 UM 8).

Therefore, there is a sense of team and reciprocity when volunteers work alongside the staff, ultimately allowing Namaste Care sessions to run more efficiently. The volunteer coordinator at one site expressed:

*“Our staff are really thrilled with our volunteer participation because the more volunteers helping out with the program, the more people that can come out and attend the programs. Because you’ve got those extra hands to help with more residents. So, the attendance is higher if there’s more volunteers. I know people [get] more attention if there’s more volunteers assisting with programs because they can sit with people during programs. They can assist them more individually…the volunteer can comfort…other residents as well who need that one-on-one attention…we really rely on the help of volunteers”* (1 UM 8).

The involvement of more volunteers may be especially helpful as not only do they have the potential to build ‘workforce’ capacity for Namaste Care, but LTC staff noted that volunteers inherently *“help a lot. It gives [residents] somebody else that they don’t see every single day come in to visit them. Somebody new to talk to that has different interests than a lot of the people here”* (1LTCS 2 Focus Group), thus making each volunteer’s contribution all the more meaningful.

*Encouraging family involvement.* All staff emphasized that it is crucial to make the Namaste Care program an open program that families feel comfortable being a part of. Thus, staff recommended that Namaste be a program that actively encourages family members to be involved, which aligns with their overall perceptions of involving families in Namaste Care:

*“I know a lot of [family members] don’t know about [Namaste], because…I’ve said [to family members], oh no you can sit in with them, go ahead. Try to engage [families] in that way and let them know. I think that would be helpful and just encourage them to come or if they have ideas. Things that maybe they want to present or participate in…instead of just getting consent for them to come. Just asking would you like to be involved? Do you want to sit in with your mom or loved one”* (1 UM 10).

This collaboration can have a powerful impact that motivates families to participate and develop stronger relationships with care home staff. A management staff member summarizes this relationship as the following:

*“[Namaste Care] does help with families and their perception that we’re doing everything that we can. And that perception is really important in the day to day. It builds the trust bank that we need to have with families. I think it can be a source of pride for the organization that we tried something innovative and different and new and we’ve stuck with it and figured it out and didn’t say no. We didn’t say not anymore. And that we’re always trying to change and grow and learn”* (1 UM 6).

Therefore, the family engagement in Namaste not only motivates future participation, but it importantly builds the trust family members have in the home taking action to address the gap in quality of care for residents with advanced dementia. As a result of this trust, some families also used their time at a Namaste session as respite so that they may take a break from monitoring or advocating. LTC staff also seemed to encourage this collaboration and recommended that measures to bolster strong communication between the LTC home and families should be implemented to facilitate their participation in the Namaste Care program. However, clinical staff noticed that families would sometimes hesitate to join the Namaste sessions in fear of interrupting, which staff recommended should be mitigated by providing encouragement and reassurance for family participation early on.

Participants consistently commented that Namaste Care was an appropriate and helpful way to facilitate partnerships in care. Being able to spend time co-delivering care allowed family caregivers and staff members to exchange observations and information they might not otherwise have had time to do. For instance, staff perceived that families participating in Namaste Care *“may be able to provide an explanation or context to feelings that a resident with advanced dementia cannot express or explain”* (1LTCS 2 Focus Group). One nurse stated that she was glad to be able to receive information from the family that she could then use to *“provide content and more information to staff about certain [responsive] behaviours”* (1NUR 3 Focus Group). Other staff members commented on how the reciprocal exchange of information generally helped to optimize residents’ care:

*“I think for certain programs too it’s really helpful when the families are there because they can engage them [the residents]. They sometimes listen to them better or they’re able to get them more active than we are at times…it’s really great to see and I think the family really enjoys it as well that we keep them up to date and really let them know what’s going. Because they really like to be engaged in those kinds of things. They look forward to going on outings with them and you know, still being able to do those types of activities…in regards to the whole communication aspect, if you are dealing with a resident who can’t articulate feelings or anything along the lines of that, we can communicate with family and figure out different things that might trigger or help in the situation at hand”* (1LTCS 4 Focus Group).

In this way, staff believed that Namaste Care is acceptable as a means to enhance partnerships between families and staff in providing care to residents. By including families in the care approach, they provide staff with valuable insights about the resident’s behaviours, actions, and thoughts. This may then allow staff to adjust their care for those residents to honour their values even if they cannot express it themselves.

Managers, nursing staff, and PSW groups identified the Namaste Care program as a way of supporting family members to meaningfully connect with residents living with advanced dementia in LTC. Families were perceived by staff as having an important role in supporting the social inclusion of residents with advanced dementia. Managers also expressed the importance of facilitating the family-resident connection through Namaste Care:

*“With family, we certainly can provide that education [on] how they can have a meaningful visit when they’re coming in to visit their loved one and what kind of activities they can utilize to make it a meaningful visit. So as far as the social relationships go, I think…family members will struggle and they often will say ‘I don’t know what to say or what to do when I come in,’ and then that’s where we can provide our expertise and help them come up with something”* (2 UM 7).

This is especially important as supervisors and managers identified that very few programs are geared towards family participation with residents in LTC homes (1 UM 6). A staff member elaborated:

*“We have a family council. I don’t know if it’s a program. It’s sort of a mandated kind of engagement group that we are to have. Beyond that we probably don’t do that much [family engagement]. I think again it’s probably done more in informal ways”* (1 UM 6).

Namaste Care was perceived by staff as potentially offering a new and meaningful way for families to participate more directly in the unique community of LTC homes.

### Implementation Barriers

Despite numerous perceived benefits of Namaste Care for residents, families, and staff, as well as many facilitators supporting program implementation, there were some barriers described by participants. These were: (a) low staffing ratios and (b) timing of Namaste Care sessions for families.

*Low staffing ratios.* While the nursing staff had positive impressions of Namaste Care, they were also initially apprehensive of the additional workload the program would create for staff. In the early implementation of Namaste Care, nurses collectively agreed that additional staff may be necessary to deliver the program:

*“Staffing definitely would not be able to take this on…we will support it…[but] the only way it could potentially happen…[is] say if you could add a part-time staff…to come in specifically for Namaste Care and you had two or three PSWs that were being paid to come in and provide this care”* (2NUR 2 Focus Group).

PSWs also agreed that Namaste Care could improve the quality of life of residents with advanced dementia, but that the program should not rely on PSWs who were already responsible for meeting the usual care needs of residents. One PSW described:

*“I feel like when you’re pulling us from the floor too, as much as I like that one-on-one* *interaction, my mind is still running and I’m saying okay I’m taking half an hour off, look at all this stuff I’ve got to go back [to] now” (1PSW 4 Focus Group).*

Management was also concerned with this issue, with one administrative staff member remarking:

*“Not to implement [Namaste Care] so much, but to sustain it – it’s been hard. It’s just getting on top of it for staffing. Who is going to run the program? The switches in schedules and times. Getting people there…pulling [staff] off the floor I know is only a half an hour [at a time], but pulling them off the floor when we’re already so stretched. Like we’re already thin as it is, right…I don’t want to pull them away if we are already seriously understaffed”* (1 UM 10).

Despite staff concerns regarding workload conflicting with the implementation of the program, homes were still able to deliver Namaste and provide dedicated time for staff to participate (aforementioned facilitator) by involving staff from various disciplines and assigning roles to lessen the burden of program delivery. A simple example such as transporting residents to the Namaste Care room exemplifies this: *“Everyone is kind of used to pitching in and getting people to where they need to go…we can make sure there’s volunteer support and that sort of thing”* (2 UM 4).

*Timing of Namaste Care sessions for families.* Namaste Care was delivered at fixed times in the morning and afternoon in the LTC homes. This made it difficult for family members who were not available at those times to attend. It was said that *“a lot of times [Namaste] is in the morning…a lot of the family members, they have young families. They work. They cannot be here”* (1PSW 4 Focus Group). Therefore, the nursing staff recommended that the time and setting of the Namaste Care program should be fluid to meet the individual needs of residents living with dementia, as well as those of their family members. In particular, some clinical staff recommended that Namaste Care sessions be delivered in the evenings and weekends, when families are presumably more likely to be available to attend. However, with evening sessions, this also raised additional challenges, as staff perceived that:

*“With the evening activities you can’t get anybody to go right? They are ready for bed…The activity girls, by the time they come, [the residents] are all in bed. Because everybody wants to go to bed, and I don’t blame them”* (1PSW 4 Focus Group).

However, even if families were able to attend, some staff believed that the length of one session was too short for family members to justify the commute. As a community centre staff explained:

*“With the Namaste Program…it’s such a small window. It’s only half an hour in the morning and then half an hour in the evening, or afternoon. So it would be hard [for families] to get out, come here for the half an hour, and then back to what you were doing before”* (1LTCS 2 Focus Group).

Therefore, despite staff emphasizing the importance of family engagement in Namaste Care, they recognized that the timing and duration of these sessions may not align with what family members are able to commit.

## Discussion

This study aimed to highlight LTC staff perspectives following implementation of the Namaste Care program in LTC—a program encompassing person-centered care for residents with dementia by giving them comfort and meaningful occupation, while simultaneously facilitating attachment, inclusion, and identity [34]. Facilitators and barriers of the program were explored to provide insight on how it can be sustainably implemented.

To elaborate on these findings, it is crucial to explore them within the context of existing literature and highlight unique contributions. In particular, there is an important need to focus on the suitability of Namaste Care for LTC residents with advanced dementia, the potential for family members to be further engaged and supported through Namaste Care, and strategies to promote facilitators and overcome barriers when implementing Namaste Care on a wider scale in Canada.

### The need that residents, families, and staff have for programs like Namaste Care in LTC homes

LTC staff identified an organizational need for the implementation of the Namaste Care program to support the increasing number of residents living with advanced dementia. They explained that current programs are not designed for persons with advanced dementia, but that Namaste Care has the potential to meet this need, in particular, benefiting the “hallway residents” who are provided with limited engagement opportunities and consequently spend the majority of their time with limited social interactions. Although Namaste Care was identified as a need by PSWs and LTC staff, the current time and workload constraints of LTC were reported as making it difficult to engage or socialize with residents in a meaningful way. This limitation for PSWs and other LTC staff has been reported in previous research, as PSWs report having the will, but not the means, to provide high quality care [35]. However, further research is needed to investigate the impact of Namaste Care on the quality of work-life and role descriptions of PSWs and LTC staff.

Staff from all occupations further noted the importance of Namaste Care in addressing residents’ needs for social engagement, as well as its ability to engage families and staff to ensure a holistic and sustainable approach to care. In this way, the Namaste Care program cannot be viewed as an independent tool to improve social interactions of residents with advanced dementia without considering how it also engages the family and staff. This is especially important as family members do not forfeit the role of a caregiver once their relative enters LTC, and continue to hold numerous key responsibilities in the resident’s care plan, such as hands-on assistance, care management, socioeconomic support, and contributions to the resident’s LTC community at large [36, 37]. Yet, their engagement should not be merely viewed as “another helping hand” for staff, as family involvement has been shown to be embedded within the complexities of the family-resident dynamic [36]. Consistent with the staff perspectives in this study, other studies on Namaste Care also suggest benefits of the program on the social relationships between residents living with advanced dementia in LTC and their family carers [12, 38]. Furthermore, early research demonstrated that families using Namaste Care to support their relatives living with advanced dementia experience an increase in quality of life measured through the GAIN (Gain in Alzheimer Care) Instrument [27]. However, such results have been preliminary and call for further investigation into the benefits of Namaste Care for family caregivers. There is a strong need to conduct this research, as family carers of residents living with advanced dementia in LTC often feel a sense of loss, and thus are also vulnerable to social isolation and consequently, a lower quality of life [18, 19].

Thus, Namaste Care can be seen as a medium and facilitator for these family roles and relationships to take place. By allowing engaged family carers to share key information about the resident’s habits and values with staff, Namaste Care can be an effective person-centered program by bridging resident needs with family involvement and staff empowerment—a relationship triad which serves as a powerful facilitator to quality resident care [39].

### Recommendations from staff for implementing Namaste Care in LTC

When considering the implementation of the Namaste Care program in LTC, different staff roles (management, nursing, PSWs, and LTC staff) provided various concerns and recommendations. Home management identified awareness of Namaste for staff and families as the primary factor for the implementation of Namaste Care in LTC. This recommendation has been supported by research demonstrating adequate training and education as a facilitator for the successful implementation of any intervention in LTC [40], which is particularly important as staff time is scarce and thus using success strategies should be prioritized. In another supporting example, a cross-sectional study in Sweden showed that care homes with leaders engaged in staff knowledge, professional development, and team support were consistently observed to have high levels of person-centered care [41].

Staff from other occupations echoed this idea for education, and recommendations were further made to conduct the training “in-house” and include family carers and volunteers. Regarding volunteer engagement, there was considerable mention of recruiting volunteers to assist with social programs like Namaste Care, with most staff supporting their involvement. This theme is particularly interesting as it builds on staff’s perceptions that with their high workloads, volunteers may be an invaluable resource to assist with the implementation of social engagement programs, which can include Namaste Care [39]. Although there were some doubts finding or having enough volunteers in such a role, the majority of participants found it appropriate and further recommended their help. However, volunteer sentiments and how volunteers may form connections with residents, are not well-understood. A systematic review of the impact of volunteering in LTC did offer a hopeful outlook on the benefit of volunteers in LTC social programs, as it was found that the involvement of volunteers in one-on-one activities improved resident engagement and mood, with most of the benefits observed during the activity itself [42]. As the review suggested, this may indicate that volunteering provided benefits for residents as they were “in the moment” of the activity—this is significant as it aligns with research describing moments of connection being of great importance for people with dementia [43]. Nevertheless, many hopeful benefits from volunteer assistance in Namaste Care, such as reducing staff workload, retaining positive long-term volunteer impact, and saving costs that can be reinvested in other areas of the home, remain unclear [42]. Additionally, it may be valuable to broaden the research on volunteers in LTC to include other types of voluntary support, such as exploring how students from health and social disciplines can participate in Namaste Care as part of courses or projects with community service learning components [39].

In addition to our discussion on the benefits of involving family members in Namaste Care, a point of interest raised by staff was that engaging families may help them better understand the value and benefits of the program first-hand. As such, Namaste Care may promote family participation, trust with staff, and even comfort for families knowing that a beneficial program exists to support their relative’s psychosocial needs. Among these potential benefits, trust among families and staff has been documented as a crucial element of collaborative family-staff relationships [44–48]. It has been reported that for many family members who partner in the care of their relative, their vigilance and observations were linked to their trust and confidence in staff [49]. In addition, family collaboration encourages shared responsibilities and seeing situations from staff’s perspectives, which has been observed to also develop trust, so long as it did not induce conflict [45, 46, 36]. Thus, Namaste Care may serve as a program medium that facilitates such relationships between staff and families.

However, it was also found that weekday morning and afternoon Namaste sessions may not be accessible for families who have jobs and other family members to support. Unlike regular visits, a session for Namaste requires more preparation in advance, involvement from staff, and rigid timelines/durations. As a result, it may be less flexible for caregivers to choose when they could attend a session with their relative. Although changing the times that the program is offered may resolve this issue for some families, it will inevitably create new timing issues for other families. While offering the program all days of the week from morning to evening would be difficult for an LTC home to achieve, it may be worthwhile for each home to conduct an analysis with their caregiver community to determine the times when most interested caregivers are available. In addition to the discussion on providing Namaste Care at a time convenient for families, an often forgotten consideration is at what times Namaste can be used to benefit residents the most. This is an area that should be valued and explored further.

### Implications for Namaste Care Implemented in LTC

Over the last two decades, Namaste Care has evolved into a tailorable group or individual program that can be offered to older adults, in any setting, who are experiencing social isolation [22]. Furthermore, Namaste Care has shown benefits and a strong alignment with a resident-centered palliative approach to care for residents living with advanced dementia, which is important throughout the entire illness trajectory including at the end of life [40]. This study demonstrates that staff often believe in the benefits of person-centered care programs that promote social engagement, like Namaste Care, and have the willingness to participate in such approaches to care but are often prevented from doing so due to their limited time and stressful workloads. This calls for homes interested in bolstering social engagement to not only include programs like Namaste Care as a mere “additional program” offered, but to also build it as a standardized program such that the structure and capacity to deliver it is sustainably provided.

As protected staff time may be difficult to delegate due to the staffing constraints in the LTC sector, volunteers have continually been mentioned by staff in this study as facilitators for Namaste Care. Recognizing this crucial theme, future research should explore the role of volunteers in supporting the implementation of Namaste Care without taking away LTC staff from the forefront of program delivery. This is especially important as volunteers should not be used to replace staff as they have professional responsibilities to ensure that all the needs of residents are being met, including social needs. Furthermore, there is a need to build LTC staff capacity in supporting the social inclusion of residents. Delegating programs such as Namaste Care to volunteers risk minimizing the importance of meaningful engagement with residents for LTC staff. Additionally, family member participation in Namaste Care should not only be an option, but a priority for LTC homes. The benefits in this involvement are far-reaching, from building program capacity to facilitating trust among staff and families, creating more personalized spaces and tailored care.

### Strengths and Limitations

The large sample size and diversity of staff were strengths in our study, allowing us to describe staff perspectives on Namaste Care from an interdisciplinary point of view. Staff with different roles in LTC settings interact with residents in unique ways—gathering these diverse thoughts allowed us to evaluate Namaste Care at a profound level.

Although this study recruited a sizable sample overall, the participants were divided further into groups based on their occupations. Thus, the reporting of certain themes that were specific to an occupation or role (i.e., management, nursing, PSW, LTC staff) were done so with only a smaller sub-sample that could limit the transferability of the results. Some staff members were also contacted for participation based on the recommendation of the home’s leadership team; thus, the selection process may have been biased. However, this did ensure that those who participated were experienced with Namaste Care. Finally, the two care homes that were involved in this study were limited to one region in Ontario, Canada, and both shared positive working relationships with the research team from previous collaborations. Consequently, the staff recruited may have been particularly enthusiastic about the intervention, and thus, reporting more positively about Namaste Care. This level of buy-in and willingness to participate may not be transferable to other care homes, illustrating the need to investigate the perception of Namaste Care among diverse LTC home settings.

## Conclusions

Overall, findings from this study bring to light the perspectives of various LTC staff groups on the implementation of the Namaste Care program as a person-centered approach to care in LTC. This study contributes to current LTC and dementia care research by highlighting the organizational needs and impressions, as well as implementation strategies for programs such as Namaste Care that facilitate social connections and meaningful engagement for residents living with advanced dementia.

We have offered some recommendations for the shift from task-oriented to person-centered care using Namaste Care as a medium. We have also shown that with family involvement in Namaste Care, the program can go beyond resident benefits to create positive care partnerships between family caregivers and staff. Future research should explore the impact of the program on the quality of life of family carers who have relatives with dementia living in LTC and the feasibility and effects of involving volunteers to facilitate the program. Further, the impact of Namaste Care on the quality of work-life and role of PSWs and LTC staff, as well as the impact of providing social engagement programs like Namaste Care at purposeful times based on factors such as a resident’s mood and routine, should be explored.

## Data Availability

The data for this research consists of questionnaires, interviews, focus groups, transcriptions, and notes. Raw data cannot be publicly released due to the risk of compromising participant confidentiality but are available from the corresponding author on reasonable request.

## References

[1] Alzheimer’s Society of Canada. Navigating the Path Forward for Dementia in Canada: The Landmark Study Report #1. 2022 (accessed 11 Oct2022). Available from http://alzheimer.ca/en/research/reports-dementia/landmark-study-report-1-path-forward.

[2] Canadian Institute for Health Information. Access to Palliative Care in Canada, Ottawa ON. ; 2018 (accessed 6 Apr2022). Available from https://www.cihi.ca/en/access-to-palliative-care-in-canada.

[3] Alzheimer’s Society of Canada. Prevalence and Monetary Costs of Dementia in Canada. Reports on dementia. 2016 (accessed 11 Oct2022). Available from https://alzheimer.ca/en/research/reports-dementia.

[4] National Seniors Council. Report on the Social Isolation of Seniors, 2013–2014. 2014 (accessed 11 Oct2022). Available from https://www.canada.ca/en/national-seniors-council/programs/publications-reports/2014/social-isolation-seniors.html.

[5] Chamberlain SA, Duggleby W, Teaster PB, Estabrooks CA (2020). Characteristics of socially isolated residents in long-term care: a retrospective cohort study. Gerontol Geriatr Med.

[6] Government of Canada. Social isolation of seniors - Volume I: Understanding the issue and finding solutions. 2017 (accessed 11 Oct2022). Available from https://www.canada.ca/en/employment-social-development/corporate/partners/seniors-forum/social-isolation-toolkit-vol1.html.

[7] National Academies of Sciences, Engineering, and Medicine (2020). Social isolation and loneliness in older adults: Opportunities for the Health Care System.

[8] Blazer D (2020). Social isolation and loneliness in older Adults—A Mental Health/Public Health Challenge. JAMA Psychiatry.

[9] Holt-Lunstad J (2017). The potential Public Health relevance of social isolation and loneliness: prevalence, epidemiology, and risk factors. Public Policy Aging Rep.

[10] Santini ZI, Jose PE, Cornwell EY, Koyanagi A, Nielsen L, Hinrichsen C (2020). Social disconnectedness, perceived isolation, and symptoms of depression and anxiety among older Americans (NSHAP): a longitudinal mediation analysis. Lancet Public Health.

[11] Alzheimer’s Society of Canada. The stages of Alzheimer’s disease. 2008 (accessed 11 Oct2022). Available from http://alzheimer.ca/en/about-dementia/what-alzheimers-disease/stages-alzheimers-disease.

[12] Manzar B, Volicer L (2015). Effects of Namaste Care: pilot study. Am J Alzheimer’s Dis.

[13] Volicer L, Simard J, Pupa JH, Medrek R, Riordan ME (2006). Effects of continuous activity programming on behavioral symptoms of dementia. J Am Med Dir Assoc.

[14] Park C, Kim D, Briesacher BA (2021). Association of Social isolation of Long-term Care Facilities in the United States with 30-Day mortality. JAMA Netw Open.

[15] Kovach CR, Magliocco JS (1998). Late-stage dementia and participation in therapeutic activities. Appl Nurs Res.

[16] Zwakhalen SMG, Koopmans RTCM, Geels PJEM, Berger MPF, Hamers JPH (2009). The prevalence of pain in nursing home residents with dementia measured using an observational pain scale. Eur J Pain Lond Engl.

[17] Statistics Canada, Caregivers. in Canada, 2018. 2018 (accessed 14 July2022). Available from https://www150.statcan.gc.ca/n1/daily-quotidien/200108/dq200108a-eng.htm.

[18] Adelman RD, Tmanova LL, Delgado D, Dion S, Lachs MS (2014). Caregiver burden: a clinical review. JAMA.

[19] Chan D, Livingston G, Jones L, Sampson EL (2013). Grief reactions in dementia carers: a systematic review. Int J Geriatr Psychiatry.

[20] Boamah SA, Weldrick R, Lee T-SJ, Taylor N (2021). Social isolation among older adults in long-term care: a scoping review. J Aging Health.

[21] Simard J (2013). The end-of-life Namaste Care Program for People with Dementia.

[22] Namaste Care. The Namaste Care Individual Program. 2020 (accessed 11 Oct2022). Available from https://namastecare.com/namaste-care-individual-program/.

[23] Stacpoole M, Hockley J, Thompsell A, Simard J, Volicer L (2015). The Namaste Care programme can reduce behavioural symptoms in care home residents with advanced dementia. Int J Geriatr Psychiatry.

[24] Stacpoole M, Hockley J, Thompsell A, Simard J, Volicer L (2017). Implementing the Namaste Care Program for residents with advanced dementia: exploring the perceptions of families and staff in UK care homes. Ann Palliat Med.

[25] Simard J, Volicer L (2010). Effects of Namaste Care on residents who do not benefit from usual activities. Am J Alzheimers Dis Other Demen.

[26] Kaasalainen S, Hunter PV, Dal Bello-Haas V, Dolovich L, Froggatt K, Hadjistavropoulos T (2020). Evaluating the feasibility and acceptability of the Namaste Care program in long-term care settings in Canada. Pilot Feasibility Stud.

[27] El Alili M, Smaling HJA, Joling KJ, Achterberg WP, Francke AL, Bosmans JE (2020). Cost-effectiveness of the Namaste care family program for nursing home residents with advanced dementia in comparison with usual care: a cluster-randomized controlled trial. BMC Health Serv Res.

[28] Kaasalainen S, Hunter PV, Hill C, Moss R, Kim J, van der Steen JT (2019). Launching ‘Namaste Care’ in Canada: findings from training sessions and initial perceptions of an end-of-life programme for people with advanced dementia. J Res Nurs JRN.

[29] Brannelly T (2011). That others Matter: the Moral Achievement—Care Ethics and Citizenship in practice with people with dementia. Ethics Soc Welf.

[30] Bradshaw C, Atkinson S, Doody O (2017). Employing a qualitative description Approach in Health Care Research. Glob Qual Nurs Res.

[31] Yous M-L, Hunter PV, Coker E, Fisher KA, Nicula M, Kazmie N, et al. Feasibility and Effects of Namaste Care for Persons with Advanced Dementia in Canadian Long-Term Care Homes. J Am Med Dir Assoc. 2023. S1525-8610(23)00422-X. 10.1016/j.jamda.2023.04.031 37301225

[32] Vaismoradi M, Turunen H, Bondas T (2013). Content analysis and thematic analysis: implications for conducting a qualitative descriptive study. Nurs Health Sci.

[33] Lincoln YS, Guba EG, Pilotta JJ (1985). Naturalistic Inquiry.

[34] Fazio S, Pace D, Flinner J, Kallmyer B (2018). The Fundamentals of person-centered care for individuals with dementia. Gerontologist.

[35] Beck I, Törnquist A, Broström L, Edberg A-K (2012). Having to focus on doing rather than being-nurse assistants’ experience of palliative care in municipal residential care settings. Int J Nurs Stud.

[36] Puurveen G, Baumbusch J, Gandhi P (2018). From family involvement to family inclusion in nursing home settings: a critical interpretive synthesis. J Fam Nurs.

[37] Hunter PV, Ward HA, Puurveen G (2023). Trust as a key measure of quality and safety after the restriction of family contact in canadian long-term care settings during the COVID-19 pandemic. Health Policy Amst Neth.

[38] Nicholls D, Chang E, Johnson A, Edenborough M (2013). Touch, the essence of caring for people with end-stage dementia: a mental health perspective in Namaste Care. Aging Ment Health.

[39] Pereira RF, Myge I, Hunter PV, Kaasalainen S (2022). Volunteers’ experiences building relationships with long-term care residents who have advanced dementia. Dement Lond Engl.

[40] Hunter PV, Kaasalainen S, Froggatt KA, Ploeg J, Dolovich L, Simard J (2017). Using the ecological framework to identify barriers and enablers to implementing Namaste Care in Canada’s long-term care system. Ann Palliat Med.

[41] Backman A, Sandman P-O, Sköldunger A (2021). Characteristics of nursing home units with high versus low levels of person-centred care in relation to leadership, staff- resident- and facility factors: findings from SWENIS, a cross-sectional study in Sweden. BMC Geriatr.

[42] Handley M, Bunn F, Dunn V, Hill C, Goodman C (2022). Effectiveness and sustainability of volunteering with older people living in care homes: a mixed methods systematic review. Health Soc Care Community.

[43] Bunn F, Lynch J, Goodman C, Sharpe R, Walshe C, Preston N (2018). Improving living and dying for people with advanced dementia living in care homes: a realist review of Namaste Care and other multisensory interventions. BMC Geriatr.

[44] Bauer M, Fetherstonhaugh D, Tarzia L, Chenco C (2014). Staff-family relationships in residential aged care facilities: the views of residents’ family members and care staff. J Appl Gerontol off J South Gerontol Soc.

[45] Wilson CB, Davies S, Nolan M (2009). Developing personal relationships in care homes: realising the contributions of staff, residents and family members. Ageing Soc.

[46] Lau W-YA, Shyu Y-IL, Lin L-C, Yang P-S (2008). Institutionalized elders with dementia: collaboration between family caregivers and nursing home staff in Taiwan. J Clin Nurs.

[47] Legault A, Ducharme F (2009). Advocating for a parent with dementia in a long-term care facility: the process experienced by daughters. J Fam Nurs.

[48] O’Shea F, Weathers E, McCarthy G (2014). Family care experiences in nursing home facilities. Nurs Older People.

[49] Baumbusch J, Phinney A (2014). Invisible hands: the role of highly involved families in long-term residential care. J Fam Nurs.

